# Systemic challenges in the supply and distribution of medicines in conflict-affected areas of Mali: a qualitative study

**DOI:** 10.1080/16549716.2026.2676369

**Published:** 2026-06-03

**Authors:** Mohamed Ali Ag Ahmed, Mama Konta, Issa Coulibaly, Djeneba Diarra, Hassane Alami, Karina Kielmann, Raffaella Ravinetto

**Affiliations:** a Administration and Management of Health Services, Moncton University, Moncton, Canada; b Management, Evaluation and Health Policy Department, University of Montreal, Montreal, Canada; c Public Health Research Centre (CReSP), University of Montreal, Montreal, Canada; d Faculty of Medicine and Odontostomalogy, University of Sciences, Techniques, andTechnologies of Bamako, Bamako, Mali; e Institute for Global Health & Development, Queen Margaret University, Edinburgh, UK; f Department of Public Health, Institute of Tropical Medicine, Antwerp, Belgium; g School of Public Health, University of the Western Cape, Cape Town, South Africa

**Keywords:** Supply, medicines, pharmaceutical system, conflict-affected areas, Mali

## Abstract

**Background:**

The conflicts that have been ongoing for over a decade in different Sahel countries, including Mali, have a negative impact on the supply and distribution of medicines.

**Objective:**

This study aims to identify and analyze the key contextual challenges affecting the supply and distribution of medicines in conflict-affected areas in Mali.

**Methods:**

This study adopts a qualitative study design. First, we conducted 28 interviews with key stakeholders in the pharmaceutical sector in the Mopti and Bamako areas. Such key stakeholders included health professionals, community association members, health authorities, and humanitarian stakeholders. Second, we conducted a thematic analysis, using NVivo software for retrieving and coding the qualitative data.

**Results:**

The key contextual factors affecting the supply and distribution of medicines in the study area have been organized under four interlinked themes: (1) insecurity and instability, disrupting transport routes and supply chains; (2) systemic and market dysfunctions, generating price instability and recurrent shortages; (3) technical and infrastructural weaknesses, compromising adequate storage conditions; and (4) insufficient coordination and collaboration among the different stakeholders, which further destabilizes the already fragile pharmaceutical supply system. Together, these factors have considerably weakened the pharmaceutical supply chain’s capacity to ensure continuous and equitable access to quality-assured medicines.

**Conclusion:**

Insecurity, market disruptions, weak infrastructure, and insufficient coordination severely undermine the supply and distribution of essential medicines in the conflict-affected areas of Mali. Restoring equitable access requires a strong coordination across public, private, and humanitarian actors, sustained investment in logistics and information systems, and the strengthening of regulatory oversight during and after the conflict.

## PAPER CONTEXT

**Main findings**: Medicine procurement and distribution in conflict-affected areas face four interconnected challenges: (1) prevailing insecurity and instability disrupting transport routes and supply chains; (2) systemic and market dysfunctions generating price instability and recurrent shortages; (3) technical and infrastructural weaknesses compromising adequate storage conditions for medicines; and (4) insufficient coordination and collaboration among actors, which further destabilizes the already fragile balance of the pharmaceutical supply system.

**Added knowledge**: Very few studies have focused on assessing and understanding the obstacles to the effective distribution and supply of medicines in conflict zones in sub-Saharan Africa. This is, to the best of our knowledge, the first study conducted in Mali to address this specific issue in conflict areas.

**Global health impact on policy and action**: This study provides new information on medicine distribution and supply to international readers of Global Health Action, health authorities, and humanitarian actors in Mali and in the other Sahel countries that have been facing unprecedented conflicts for over a decade. The findings highlight the determinants of the problem, which is a first essential step towards proposing measures to improve access to healthcare for populations in conflict areas.

## Background

A strong political commitment for universal access to essential medicines was first enshrined in the Alma-Ata Declaration on Primary Health Care (1978), and then reaffirmed in the Astana Declaration (2018) [1], and consolidated through the Sustainable Development Goals [2,3]. Nevertheless, the World Health Organization (WHO) estimates that nearly two billion people still lack access to essential medicines [4]. A major obstacle to universal health coverage (UHC) is the fragility of medicines supply and distribution systems, which leads, particularly in many low- and middle-income countries (LMICs), to frequent shortages and stock-outs [5–8]. This fragility is exacerbated during armed conflicts: the health needs increase [9–11], but the collapse of supply and distribution chains compromises the supply of quality-assured medicines, particularly in the most remote areas [10–12]. However, accurate data on the performance of pharmaceutical systems in conflict settings are still limited, due to the logistic and security challenges which discourage on-site data collection and to the limited interest from most research funders.

Mali, one of the poorest countries in the world [13], is a case in point. It covers an area of approximately 1,241,238 km^2^, with a population of 20,933,072 (in 2020). The country faces enormous public health challenges, with more than 70% of the burden of disease linked to infectious, neonatal, maternal, and nutritional diseases [14]. Recent estimates (2023) suggest that maternal mortality has declined to approximately 317–367 per 100,000 live births, while infant mortality remains high at around 58 per 1,000 live births. However, these national averages may mask significant disparities in conflict-affected regions [15]. Timely access to the medicines needed to address this burden is compromised by a decade-long security crisis, involving armed groups with territorial claims and terrorist factions [16–18]. The growing insecurity has led to massive population displacements [19]; as of August 2025, there were an estimated 334,000 refugees and 402,000 internally displaced persons (IDPs) [20]. The military coups of 2020 and 2021 and the shaky political transition process thereafter have added on to the general instability. In 2022, an estimated 7.5 million people (i.e. more than 1 in 3 Malians) required humanitarian aid, with an increase of 20% since 2021 [20].

The political and military crisis has profoundly affected the health system: by 2015, around 294 of 1,581 health facilities had been destroyed or damaged (18.6%) [21]. The ongoing conflict caused the destruction of infrastructure, looting of equipment, attacks on healthcare personnel, closure of health centres, exodus of healthcare professionals, and disruptions in the supply of medicines and medical equipment, in line with what is observed in other conflicts [10,22,23].

The pharmaceutical sector is particularly vulnerable to the effects of conflict, due to the pre-existing weak regulatory oversight [24]. It comprises different structures at different levels. At the central level, there are the *Pharmacie Populaire du Mali* (PPM), responsible for purchasing and distributing medicines, and the *Direction de la Pharmacie et des Médicaments* (DPM), which is the regulatory authority. The PPM and the private wholesalers import the medicines and distribute them to the regional warehouses. These supply the district distribution depots, which in turn supply the sales depots in the Reference Health Centres and Community Health Centres [25]. The private network of dispensaries and sales depots, supplied by private wholesalers, completes the system. Major and often interrelated system flaws include the limited capacity for pharmaceutical quality assurance and quality control, the presence of a large informal market, the frequent shortages and stock-outs, and the health workforce shortages [26,27].

Cost recovery has been in place since the launch of Bamako initiative [28]. The Community Health Centres and Reference Health Centres sell medicines, and a portion of the generated revenue is used to replenish the stocks. The system is managed by the local management committees, provided with standardized management tools. The health staff and the members of the Community Health Associations (*ASACO)* should, in principle, be trained for these tasks. The Ministry of Health sets the profit margin for each medicine at each level of care. Medicines are transported from central to district level by PPM vehicles and, occasionally, by private companies.

This system had been designed to guarantee a national supply of medicines under normal conditions and has been seriously disrupted by the armed conflict [26]. For instance, in conflict-affected areas, disruptions now most critically occur at the regional and district levels, where insecurity hinders the transport of medicines from regional warehouses to district depots, compromising the last-mile distribution to health facilities. The reduction in international funding and the restriction of humanitarian spaces have exacerbated shortages and stock-outs [29,30], while the increasing fragmentation of the supply chain encouraged the proliferation of intermediaries and parallel circuits, threatening the affordability and quality of available medicines. Stock management remains fragile at local health facilities.

Therefore, this study aims to identify and analyze the key contextual challenges affecting the supply and distribution of medicines in conflict-affected areas in Mali.

## Methods

### Study design and setting

We adopted a qualitative study design, using semi-structured interviews to explore the perceptions of stakeholders at the three levels of the pharmaceutical sector (operational, regional, and central) on challenges related to medicine distribution and supply in conflict-affected areas [31,32]. Emphasis was placed on eliciting informants’ opinions, beliefs, and motivations [33,34] related to the challenges faced by the Malian pharmaceutical system.

The study was conducted in the Mopti region and in the capital city, Bamako. *Mopti* region was selected because, even if it is the epicentre of the conflict, it is easily accessible by air from Bamako. With a surface area of 79,017 km^2^ and an estimated population of 3,041,000 in 2022, the region is divided into eight health districts and 108 communes. Within the four health districts (Djenné, Bandiagara, Koro, and Mopti), we selected health areas that were less than two hours’ drive from the district capital. This selection was guided by security concerns for the field researchers, as well as by the costs associated with long-distance travel. The *Bamak*o region was included because it hosts the central authorities and the headquarters of several international and national stakeholders. Data collection took place between September and November 2023.

### Study population, sampling, and recruitment

The study population comprised four relevant groups of stakeholders in the pharmaceutical sector: 1) health professionals; 2) members of Community Health Associations *(ASACO);* 3) health authorities; and 4) humanitarian workers (Table 1). These groups were chosen because of their role in medicines supply and distribution, their familiarity with the local context, and their diverse range of perspectives on the Malian pharmaceutical system. At the individual level, the inclusion criteria were being at least 18 years old, occupying a key role in medicines supply and distribution, being fluent in French and being willing to give free informed consent to participate. Saturation was reached after 28 interviews. Out of 28 participants, there were three women and 25 men, ranging in age from 26 to 60 years.

**Table 1.** Characteristics of the sample.

Structure/Status	Public Pharmaceutical Procurement Center (PPM)	Directorate of Pharmacy and Medicines (DPM)	Regional Health Directorate (DRS)	Reference Health Centres	Community Health Centres	Private pharmacy	Humanitarian actors	Total
Pharmacist	1	2	1	1		2	2	9
District Chief Medical Officer				2				2
Technical Director				6				6
Manager				3	5			8
Community Health Associations				1				1
Supply officer							2	2
**Total**	**1**	**2**	**1**	**6**	**12**	**2**	**4**	**28**

Sampling was purposive, guided by the principles of saturation and diversification [35]. Data saturation refers to the point at which further data collection no longer provides new or significant information [36,37]. Diversification aims to maximise the variety of perspectives, contexts, and experiences to obtain a richer and more nuanced understanding of the phenomenon under study [38].

### Data collection

Interviews were conducted at the operational and regional levels, that is, at the administrative centres and the targeted health areas of the selected districts, and at the central level in Bamako, to clarify and supplement the information gathered at the field level. The research team included both female and male researchers. Interviews were conducted by MK (research assistant) and a local interviewer with extensive experience in qualitative research. Both are natives of the Mopti region and familiar with the cultural context, which was important for building the rapport with the respondents. They received two days of training from the main investigators (MAAA, IC).

The interview guide explored the challenges experienced in supplying and distributing medicines. It was pre-tested and refined with four health professionals in the Kati health district, and with two depot managers in Mopti. The research assistant contacted potential participants individually to explain the study objectives and procedures, answer their questions, and ask if they were willing to participate. For those consenting, an interview was arranged at a convenient time and place. All interviews were conducted in French, face-to-face, at the participants’ place of work. Interviews were recorded using a voice recorder, and field notes were taken. All those who were contacted consented to participate. Interviews lasted between 29 and 79 minutes.

### Data analysis

We used thematic analysis, as proposed by Braun and Clarke [39], to identify, list, and group themes and sub-themes pertinent to our central topic. Data analysis started in the field by reviewing the field notes and listening to recordings. After verbatim transcription, the transcripts of the interviews were uploaded to Nvivo software (2023 version). Data analysis was undertaken by MK and DD under the supervision of the principal investigators (MAAA and IC). The analysis followed a three-stage inductive approach (coding, thematization, and analysis) [40]. The transcripts were examined line-by-line and paragraph-by-paragraph to generate codes in the form of labels assigned to the units of analysis. Codes were grouped under sub-themes, which were organised under a single emergent theme. This process resulted in a hierarchical thematic tree, reflecting four central and peripheral themes. The emerging thematic groupings were compared and contrasted to determine whether they converged, diverged, or complemented each other. These clusters were presented as significance matrices, highlighting and illustrating the challenges encountered in supplying and distributing medicines. A workshop was organized to present the results, and the participants feedback helped to cross-validate the information gathered from the interviews, thereby strengthening the robustness of the analysis.

## Results

Four interrelated themes emerged in relation to contextual factors negatively impacting on the supply and distribution of medicines in the conflict-affected areas of Mali:

1. *Prevailing insecurity and instability disrupting medicine distribution and supply chains*

Insecurity was described as a direct challenge along the transport routes. Stakeholders described armed attacks, blockades, and threats that made certain areas inaccessible. These obstacles resulted in frequent stockouts and significant distribution delays. As a pharmacist explained:

We can plan to bring medicines at a certain time, but in the meantime, if there are risks along the way or on other routes, we must postpone and wait for another opportunity. On the road to Gao, we have two removed vehicles that we are still trying to trace; that was towards the end of 2019. The case is still with the *gendarmerie* [police]. (Pharmacist, PPM)

Beyond logistical delays, insecurity affected territorial governance. Certain areas under the control of armed groups became administratively disconnected from formal supply circuits, forcing district managers to adapt distribution strategies. For instance, they resorted to informal or indirect channels, such as local weekly fairs, to ensure that medicines reached their destinations:

Some areas are inaccessible due to threats from armed groups, especially Koa, Moura, and Kouakourou. They were making many orders. There is also Mougna, which affects our profits and returns when buying medicines. We also base our orders on this. So, if Community Health Centres are not working, that can sometimes cause problems. (District Distribution Depot Manager)

Access must, in some cases, be renegotiated outside the formal state authority. This resulted in a shift from state governance to negotiated humanitarian spaces, accompanied by a transition of power from centralized coordination to territorially negotiated distribution systems:

The inter-community dialogue is used to negotiate passes with the various armed groups to allow medicines to be transported to the health areas. (Deputy Medical Doctor)

The massive population displacement has added a layer of ‘demographic shock’. The demand forecasting became hectic or unpredictable, with increasing pressure on facilities that were still accessible:

Everyone has left the bush to come and sit in Bandiagara, which puts pressure on the health centre … sometimes they say that even in Bamako, there is no CTA (Artemisinin-based combination therapies). (Sales Depot Manager, CHC)

1. *Systemic and market dysfunctions causing price instability and recurrent shortages*

Various stakeholders described how prolonged instability destabilized prices and weakened the trust in public procurement. For instance, the price volatility at PPM level made it increasingly difficult to adequately plan procurement at peripheral level:

The prices of medicines vary a lot, even at the PPM level … when you buy Novalgin® at 250 this quarter, next quarter you can get it at three hundred (300) or even three hundred and eighty (380), it is too much. (Pharmacist, Reference Health Centre)

This fluctuation influenced procurement behavioural incentives. When the public supply proved unreliable, local health facilities were forced to diversify their sources, thereby unintentionally reinforcing the fragility of the public sector:

There are managers who prefer to increase the price … if it is 13.2 francs, the manager will sell at 15 or 20 francs. Others even sell for 25 francs. (Pharmacist, Humanitarian Actor)

Confronted with price instability and recurrent shortages, many CHCs turned to private wholesalers. Again, this allowed them to address shortages in the short term, but undermined the public supply chain in the long term.

Few Reference Health Centres buy their supplies exclusively from PPM because often, out of ten products ordered, we may only have three or four. So, the customer may go to a competitor for the whole order. (Pharmacist, Regional PPM)

This dynamic fostered the expansion of parallel and informal markets, reshaping health-seeking pathways and exposing patients to higher financial risk:

They will treat, treat, treat, and finally, when things are not going well at all … they come to the CHC. These itinerant healers sell on credit, sometimes doubling or tripling prices. Through their fault, the patient comes to us to die. (Sales Depot Manager, CHC)

The fragility of financial governance further compounded these distortions. Due to local banking closures, in particular, cash started to circulate outside the formal channels:

We do not have a bank next to us … the bank has closed. To do any banking, you must go to Mopti. (ASACO Member)

At the central level, the state reimbursements to the PPM are delayed, hampering the PPM’s redistributive capacity. The fiscal instability causes supply unreliability. Together, these processes result in the progressive erosion of institutional coherence and in an unwanted reconfiguration of the pharmaceutical sector.

1. *Technical and infrastructural weaknesses leading to inadequate medicine storage conditions*

The quality of medicines is multifactorial and also depends on the quality of the storage and warehousing infrastructure. The conflict magnified pre-existing infrastructural weaknesses, including inadequate depots. Without ongoing maintenance, the exposure to flooding and frequent power outages compromises storage integrity and threaten the quality of medicines:

In flooded areas such as Djenné, most depots are inadequately built … During the winter, humidity can destroy medicines and affect their quality. Because of the conflict, these buildings are not being rehabilitated. (Pharmacist, Reference Health Centre)

The gaps in managerial capacity introduce another layer of vulnerability. For instance, the weak training in inventory systems hampers the reliability of stock data and negatively affects planning accuracy:

We found that most of the data on the needs expressed were incorrect … there are problems with the level of training. (Pharmacist, PPM)

Our last training session was in 2012. From 2012 to 2023, how many years is that? (Sales Depot Manager, CHC)

Such shortcomings could be corrected by adequate monitoring and supervision, but this is hampered by disruptions in supervisory plans, and even by communication sabotage:

The armed groups sabotaged the telephone networks … Sometimes, you must wake up in the middle of the night to get a signal. (Pharmacist, Humanitarian Worker)

These findings suggest a cumulative pattern of vulnerability. The technical and infrastructural weaknesses caused or exacerbated by the insecurity do not only hamper access to medicines; they cause a progressive degradation of system learning, monitoring and supervision, and quality assurance mechanisms.

1. *Inadequate collaboration and coordination among actors disrupting the fragile balance of the supply system*

Humanitarian stakeholders were seen as essential actors in a conflict context, yet their operational modalities sometimes generated structural tension within the national pharmaceutical system. In particular, the distribution of medicines for free in the health facilities or programs run by NGOs, weakened the cost-recovery mechanisms that are central to the Malian community-based financing:

The fact that NGOs are not respecting the master plan has caused us to lose many products … stocks of medicines at the district depot level are not being ordered and are going out of date. (District Distribution Depot Manager)

Even if well intentioned, this strategy created incentive asymmetries between the public facilities and the NGO-supported services, altering patient purchasing behaviour and reducing the revenue flows to CHCs. Rather than a simple ‘competition’ between sectors, the participants described a structural misalignment between the short-term humanitarian logic and the long-term system sustainability.

Overall, the supply and distribution of medicines in the conflict-affected areas of Mali are hampered by a combination of insecurity, fragmented governance, economic fragility, and poor coordination. The challenges identified in this study reflect a chain of interdependent failures: the chronic insecurity limits physical access to the health facilities; the financial instability erodes institutional trust; the disruption of training and supervision results in technical and human resource weaknesses, further reducing the system responsiveness; and the poorly coordinated external interventions unintentionally exacerbate structural vulnerabilities. Together, these dynamics weaken the public pharmaceutical system’s ability to guarantee continuous, sustained, and equitable access to quality-assured medicines, creating long-term distortions in the pharmaceutical system.

## Discussion

This study identified four interrelated themes that collectively describe and explain the weaknesses of pharmaceutical supply and distribution in the conflict-affected areas of Mali. The general insecurity disrupted transport and hampered the physical access to facilities. The market and systemic failures, including price volatility, weak regulatory oversight, and financial constraints, further destabilized supply. The logistical and infrastructural weaknesses reduced the system’s performance, while poorly coordinated humanitarian interventions increased the system fragmentation.

These findings need to be framed in the broader continental context. Africa faces a dual challenge of infectious and non-communicable diseases, and universal access to medicines is crucial to reduce morbidity and mortality [41–44]. To achieve this aim, supply and distribution systems should operate effectively [41], but the system disruptions caused by war and political instability seriously compromise both supply and access. Conflicts not only generate logistical disruption, but they also negatively reconfigure the entire pharmaceutical ecosystem.

### Conflict-driven reconfiguration of the pharmaceutical ecosystem

The prolonged conflict does not merely disrupt the Malian transport routes network; it progressively reconfigures the organization and governance of pharmaceutical supply. We do not observe a complete institutional collapse, but rather a ‘regulatory dilution’, with a hybrid ecosystem where public, private, informal, and humanitarian actors operate in overlapping spheres of authority. This distinction is critical. The PPM, that is the national procurement agency, remains operational, yet its authority and legitimacy are weakened; the system functions, but coherence declines. Similar hybridization processes have been described in Burkina Faso, Niger, and Sudan [45–47], suggesting that the multiplication of supply systems is a structural feature, rather than a temporary anomaly, in protracted conflict settings.

### Erosion of the public pharmaceutical system

The weakening of the central procurement system reflects more than operational inefficiencies. These are observed also in politically stable low-resource settings, with frequent shortages and stock-outs, caused by poor stock management and limited supervision [48–50]. In conflict settings, however, operational weaknesses are compounded by territorial fragmentation, financial instability, and disrupted oversight mechanisms. In Mali, the delivery delays and incomplete orders from the central level cause long-term modifications of procurement patterns: the peripheral facilities diversify supply sources, turning to private wholesalers, and unwantedly further weakening the public system. Similar ‘bypassing’ of the public supply channels have been documented in Sudan and Burkina Faso [44–46]. This shift responds to short-term urgent needs, but in the long term, it gradually reduces reliance on the public system and weakens the country’s resilience. The financial fragility further aggravates the situation. Bank closures and delayed reimbursements undermine the predictability of payments and of consignments, which is essential to build or maintain trust in the public system.

Noteworthy, the ‘erosion’ of the public system has not brought it to the point of collapse. The institutions do not disappear, but they lose their oversight and coordination capacity; their authority remains formally intact, yet operational performance declines, resulting in a subtler but chronic form of fragility.

### Expansion of private and informal actors

The disruption of the public supply system drives a rapid growth of the private and informal markets [47]. It is well known that weak regulatory oversight hampers adequate processes for pharmaceutical quality assurance, quality control, and traceability [44]. When poor oversight is aggravated by a conflict, = substandard, falsified, or unregistered medicines can come to represent a significant share of the market [51].

However, in the current Malian context, the informal sector cannot be understood solely as an illicit phenomenon: it often represents a pragmatic way to respond to the access barriers created by conflict and instability. Flexible payment modalities, credit arrangements, and geographical proximity represent pragmatic ways to access medicines in insecure environments. In this sense, the informal actors provide short-term accessibility, when formal systems fail. Yet this adaptive function comes at systemic costs: fragmented, unregulated sourcing blurs accountability, complicates traceability and pharmacovigilance, and significantly increases exposure to substandard or falsified medicines. The unregulated ‘market diversification’ brings both resilience and public health risks.

From a systems perspective, this dynamic illustrates a case of fragmented resilience, i.e. the capacity of individual actors to adapt autonomously, while collective governance coherence deteriorates [43,52]. Here, access to medicines is maintained through local coping strategies, rather than through institutionalized reforms that could prevent or mitigate public health risks.

### The dual role of humanitarian organizations

Medical-humanitarian actors are essential during acute crises. Under such circumstances, they provide free care – including essential medicines and services – while public systems are overstretched or collapsed [45,53]. However, our findings suggest a structural tension between emergency interventions and long-term system sustainability. The parallel supply chains and free distribution models may unintentionally weaken cost-recovery or other financing mechanisms that are central to community-based financing. The ‘competition’ between fee-based public services and free NGO-supported care alters patient flows and revenue streams, affecting in the long term its financial sustainability.

This tension is not unique to Mali. Similar overlaps between the humanitarian and state supply systems have been documented in Burkina Faso and Sudan [45]. Yet international experience also demonstrates that an adequate coordination between national systems and humanitarian actors can achieve a balance between the individual‘s right to health and the sustainability of the health system – and even transform duplication into reinforcement. For example, in South Sudan pooled procurement and shared distribution hubs reduced the system fragmentation and improved its transparency [45].

The policy issue at stake, here, is not whether humanitarian presence is needed or not, but rather how to shape the coordination architecture. Joint procurement planning, shared storage facilities, harmonized reporting systems and a constant, structural dialogue can allow humanitarian assistance to strengthen rather than substitute national systems.

### Rethinking resilience: coping versus adaptation

The term ‘resilience’ is often used to describe systems that continue functioning under stress. However, resilience modalities will vary depending on the specific context. In the case of Mali, our findings suggest that the pharmaceutical system demonstrates short-term coping or absorptive capacities, rather than adaptive transformation. Measures such as the use of weekly fairs to transport medicines, storing cash at home due to closed banks, and purchasing from private wholesalers are survival strategies that allow continuity, but do not correct structural vulnerabilities.

This pattern reflects short-term resilience, that is an ability to absorb shocks without systemic change [43]. In contrast, adaptive resilience implies institutional learning and durable reorganization and generally becomes possible in a post-conflict phase. In post-war Uganda, for instance, such a transformation happened when the health reforms in 1986 resulted in the decentralization of pharmaceutical logistics and in the reinforcement of supervision [54]. In Mali, which has not yet reached a ‘stable’ post-conflict state, adaptive learning remains limited. The different stakeholders innovate operationally but within fragile, temporary arrangements. Adaptive behaviors such as bypassing standard procurement rules, relying on informal vendors, or negotiating with armed groups, may in the long-term entrench parallel governance and erode trust. The key policy challenge here is to convert coping into learning: identifying local innovations that work and embedding them into formal procedures, rather than leaving them in the informal sphere. However, this may remain a theoretical exercise as long as the conflict continues to subject the system and the population to acute, repeated shocks.

The comparison of Mali’s findings with other experiences in the Sahel setting underscores both common patterns and context specificities. Insecurity, energy shortages, and market fragmentation are widespread also across Burkina Faso, Niger, and Sudan [45–47]. However, unlike Chad or South Sudan, Mali retains a partially functional national pharmaceutical authority. Even in a context heavily supported by humanitarian actors, the presence of partially functioning central institutions provides a critical entry point for reconstruction.

The case of Mali also shows that overlapping crises (security, political, financial, etc.) force systems to constantly adapt rather than recover in a linear fashion. The pharmaceutical sector became a microcosm of layered fragility: it is regulated by the state, driven by health needs and market forces, and *de facto* managed by humanitarian organizations. Understanding this hybrid ecosystem will be critical for designing realistic resilience and reconstruction strategies. Instead of restoring the pre-conflict monopoly of the public sector, policies should aim to govern pluralism by recognizing the role of multiple actors, with the ultimate strategic aim of ensuring accountability, quality, and equity, through stringent regulatory oversight that protects public health across all sectors.

### Strengthening resilience through contextualized strategies

Our results, as well as comparisons with data from other countries in the Sahel, suggest four interlinked priorities for strengthening pharmaceutical system resilience.

First, for humanitarian actors, coordination with the local system should go beyond information sharing. Creating common supply chains, including co-managed warehouses, would reduce duplications and ensure complementarity. Humanitarian partners could also support the national logistics and infrastructure (e.g. with solar energy systems, refrigeration, and digital inventory tools) by reinforcing rather than replacing national capacity, and by ensuring sustainability as part of a well-thought-out exit strategy.

Second, rapidly eliminating informal markets is unrealistic in conflict and post-conflict situations. Instead, there should be an effort to gradually train, correct, and integrate the informal sector into the regulated one. Tanzania’s model of accredited drug dispensing outlets could inspire a ‘prudent engagement and accreditation’ strategy [55], where selected informal sellers could be trained, licensed, and monitored, for maintaining accessibility while eliminating – or at least mitigating – risks. Such an exercise would require a stepwise approach, aiming at training and formalizing community-trusted vendors, and bringing them under regulatory oversight.

Third, in presence of frequent power cuts and network sabotage, high-tech digital solutions for stock planning and management are impractical. Low-tech and offline-compatible tools, such as tablet-based stock cards or standardized paper ledgers synchronized periodically, would be more realistic and sustainable. Training key-district staff in basic stock management and inventory could restore stock data reliability [56]. Integrating NGO and public reporting templates would improve the national stock planning, which is otherwise fragmented across different supply lines.

Last, restoring confidence in the public system requires reliable financing, transparent pricing, and stringent oversight. Stringent pharmaceutical regulation supported by an independent quality-control laboratory (in-country or via reliance on regional laboratories) would rebuild credibility and demonstrate a renewed state authority in pharmaceutical governance. However, this requires significant investments that would probably only become feasible in a post-conflict situation.

### Limitations, strengths and future research

Our study included only health facilities located within less than two hours’ drive of the main district towns, so it likely underestimates the challenges in more remote or inaccessible areas. Moreover, the actual needs may exceed those observed here, as many people cannot safely travel to formal or informal outlets, or they do not afford medicines at all. Finally, there is a clear gender imbalance among the participants, which may have affected the diversity of the perspectives gathered. Participants were selected based on their roles within the pharmaceutical system, but the relative underrepresentation of women could have narrowed the views and understandings of the systems’ fragilities.

We took several measures to enhance the rigour of the study [57]: interview guides were pre-tested and refined prior to data collection; a diverse range of stakeholders was included; consistent themes across interviews strengthened the credibility of the findings; and the preliminary interpretations were discussed within the research team and compared with existing literature to ensure coherence. Transferability was supported by a detailed description of the study setting, sampling strategy, and analytical process. Participants were selected to ensure variation, and data collection continued until thematic saturation was reached. Dependability and confirmability were reinforced through systematic coding using NVivo, iterative analysis, and team discussions to refine themes. Researchers maintained reflexive awareness of their positionality and documented key methodological decisions, ensuring transparency and traceability of the analytical process.

Future research should incorporate comparative, longitudinal, and cross-country analyses to understand how hybrid pharmaceutical systems in conflict and post-conflict contexts evolve over time, and to identify the best governance practices to achieve and sustain equitable access to medicines.

## Conclusion

The shocks undergone by medicine supply and distribution in conflict settings cause more than logistical disruption: they result in a systemic reconfiguration driven by insecurity, market fragmentation, and institutional fragility. The conflict disrupts and reshapes governance, weakens trust, and alters professional practices, producing structural imbalances that may persist beyond the end of the conflict.

To (re)achieve sustainable access to medicines for all, the system requires coordinated, context-sensitive pharmaceutical policies that reconcile the public, private, humanitarian, and informal actors. Humanitarian organizations should shift from parallel substitution models towards structured partnerships with national and local systems. International donors must support flexible, adaptive funding that strengthens regulation, local capacity, and institutional learning. Securing equitable and safe access to medicines in conflict and post-conflict contexts demands inclusive governance of an evolving pharmaceutical ecosystem, rather than isolated support that foster fragmented resilience within weakened public structures.

## Supplementary Material

COEQ GHA F.docx

## Data Availability

The data supporting the findings of this study are available upon request from the corresponding author (MAAA). The data are not publicly available because they contain information that could compromise the privacy of research participants.
