# Supplementary material for: Systemic challenges in the supply and distribution of medicines in conflict-affected areas of Mali: a qualitative study
Source: Glob Health Action. 2026 Jun 3;19(1):2676369. doi: 10.1080/16549716.2026.2676369 (PMC13235224; doi:10.1080/16549716.2026.2676369)
Supplement: COEQ GHA F.docx [file ZGHA_A_2676369_SM3095.docx]

| **Title: Challenges in Medicine Supply and Distribution in Conflict-Affected Areas of the Sahel: A Qualitative Study in Mali.** | | | |
| --- | --- | --- | --- |
| **Consolidated criteria for reporting qualitative studies (COREQ): 32-item checklist** | | | |
| **No** | **Item** | **Guide questions/description** | **Report** |
| **Domain 1: Research team and reflexivity** | | | |
| **Personal Characteristics** | | | |
| 1. | Interviewer/facilitator | Which author/s conducted the interview or focus group? | The data were collected by the second co-author and an investigator under the supervision of a researcher (IC). Lines 165-167 |
| 2. | Credentials | What were the researcher's credentials? E.g. PhD, MD | Five authors have PhDs in public health or community health (MAAA, HA, IC, KK, and RR), and two authors have master's degrees in public health. |
| 3. | Occupation | What was their occupation at the time of the study? | They are assistants or lecturers at their universities. |
| 4. | Gender | Was the researcher male or female? | The research team included female and male researchers. |
| 5. | Experience and training | What experience or training did the researcher have? | The researchers have several years of experience in qualitative research. Line 165 |
| **Relationship with participants** | | | |
| 6. | Relationship established | Was a relationship established prior to study commencement? | The researchers and interviewers contacted the participants before conducting the interviews. Line 175-177 |
| 7. | Participant knowledge of the interviewer | What did the participants know about the researcher? e.g. personal goals, reasons for doing the research | There was no personal relationship between interviewers and participants prior to data collection. Initial contact was made before the interviews. Line 173-174 |
| 8. | Interviewer characteristics | What characteristics were reported about the interviewer/facilitator? e.g. Bias, assumptions, reasons and interests in the research topic | None known. |
| **Domain 2: study design** | | | |
| **Theoretical framework** | | | |
| 9. | Methodological orientation and Theory | What methodological orientation was stated to underpin the study? e.g. grounded theory, discourse analysis, ethnography, phenomenology, content analysis | Data analysis for this manuscript was guided by a thematic analysis approach. This was specified in the article line 184-185 |
| **Participant selection** | | | |
| 10. | Sampling | How were participants selected? e.g. purposive, convenience, consecutive, snowball | Participants were recruited using purposive sampling. Line 154 |
| 11. | Method of approach | How were participants approached? e.g. face-to-face, telephone, mail, email | All interviews were conducted face-to-face, and this was specified in the article line 180 |
| 12. | Sample size | How many participants were in the study? | The article mentioned that there were 28 participants. This is specified in the article line 145 |
| 13. | Non-participation | How many people refused to participate or dropped out? Reasons? | The article mentioned that there were no refusals. Line 182-183 |
| **Setting** | | | |
| 14. | Setting of data collection | Where was the data collected? e.g. home, clinic, workplace | The article mentioned that the interviews were conducted at the participants' workplace (chosen by the participants). The locations where the data was collected are also specified. Line 180 |
| 15. | Presence of non-participants | Was anyone else present besides the participants and researchers? | No one else was present during the interviews which took place at a time chosen by the participants. Line 180 |
| 16. | Description of sample | What are the important characteristics of the sample? e.g. demographic data, date | The gender and age (oldest and youngest) as well as other characteristics (Table 1) of the participants are included in the article. |
| **Data collection** | | | |
| 17. | Interview guide | Were questions, prompts, guides provided by the authors? Was it pilot tested? | Yes. This is specified in the article. The interview guides were provided and tested before the interviews. Line 175-176 |
| 18. | Repeat interviews | Were repeat interviews carried out? If yes, how many? | No repeat interviews were carried out. |
| 19. | Audio/visual recording | Did the research use audio or visual recording to collect the data? | All interviews were audio recorded using a sound recording device and stored anonymously on the lead author's laptop. Line 181-182 |
| 20. | Field notes | Were field notes made during and/or after the interview or focus group? | Yes. Field notes were taken for the interviews. This is specified in the article. Line 187-188 |
| 21. | Duration | What was the duration of the interviews or focus group? | Yes. Their durations are specified in the article. |
| 22. | Data saturation | Was data saturation discussed? | Yes. This is specified in the article. We achieved saturation. Line 150-151 |
| 23. | Transcripts returned | Were transcripts returned to participants for comment and/or correction? | No. This was not possible due to budget constraints. However, a presentation was made during a workshop to validate the results. |
| **Domain 3: analysis and findingsz** | | | |
| **Data analysis** | | | |
| 24. | Number of data coders | How many data coders coded the data? | Data encoding was performed by two co-authors (MK and DD) under the supervision of the principal investigators (MAAA and IC). Line 190-191 |
| 25. | Description of the coding tree | Did authors provide a description of the coding tree? | Yes. This is specified in the data analysis |
| 26. | Derivation of themes | Were themes identified in advance or derived from the data? | YES. An inductive approach was used. This is specified in the article Line 192 |
| 27. | Software | What software, if applicable, was used to manage the data? | YES. Nvivo software was used. This is specified in the article line 190 |
| 28. | Participant checking | Did participants provide feedback on the findings? | Yes. A workshop was organized to present the results, during which some participants were able to give their feedback. Line 200-201 |
| **Reporting** | | | |
| 29. | Quotations presented | Were participant quotations presented to illustrate the themes / findings? Was each quotation identified? e.g. participant number | Yes. |
| 30. | Data and findings consistent | Was there consistency between the data presented and the findings? | Yes |
| 31. | Clarity of major themes | Were major themes clearly presented in the findings? | Yes. |
| 32. | Clarity of minor themes | Is there a description of diverse cases or discussion of minor themes? | Yes |

Developed based on: Tong A, Sainsbury P, Craig J. Consolidated criteria for reporting qualitative research (COREQ): a 32-item checklist for interviews and focus groups. International Journal for Quality in Health Care. 2007. Volume 19, Number 6: pp. 349 – 357
